# Impact of adjuvant: Trivalent vaccine with quadrivalent-like protection against heterologous Yamagata-lineage influenza B virus

**DOI:** 10.3389/fimmu.2022.1002286

**Published:** 2022-09-30

**Authors:** Mallory L. Myers, John R. Gallagher, De’Marcus D. Woolfork, Regan K. Stradtmann-Carvalho, Samantha Maldonado-Puga, Kevin W. Bock, Seyhan Boyoglu-Barnum, Hubza Syeda, Adrian Creanga, Derron A. Alves, Masaru Kanekiyo, Audray K. Harris

**Affiliations:** ^1^ Structural Informatics Unit, Laboratory of Infectious Diseases, National Institute of Allergy and Infectious Diseases, National Institutes of Health, Bethesda, MD, United States; ^2^ Infectious Disease Pathogenesis Section, National Institute of Allergy and Infectious Diseases, National Institutes of Health, Bethesda, MD, United States; ^3^ Vaccine Research Center, National Institute of Allergy and Infectious Diseases, National Institutes of Health, Bethesda, MD, United States

**Keywords:** influenza B, MF59 adjuvant, commercial vaccine, challenge, Yamagata lineage

## Abstract

As new vaccine technologies and platforms, such as nanoparticles and novel adjuvants, are developed to aid in the establishment of a universal influenza vaccine, studying traditional influenza split/subunit vaccines should not be overlooked. Commercially available vaccines are typically studied in terms of influenza A H1 and H3 viruses but influenza B viruses need to be examined as well. Thus, there is a need to both understand the limitations of split/subunit vaccines and develop strategies to overcome those limitations, particularly their ability to elicit cross-reactive antibodies to the co-circulating Victoria (B-V) and Yamagata (B-Y) lineages of human influenza B viruses. In this study, we compared three commercial influenza hemagglutinin (HA) split/subunit vaccines, one quadrivalent (H1, H3, B-V, B-Y HAs) and two trivalent (H1, H3, B-V HAs), to characterize potential differences in their antibody responses and protection against a B-Y challenge. We found that the trivalent adjuvanted vaccine Fluad, formulated without B-Y HA, was able to produce antibodies to B-Y (cross-lineage) on a similar level to those elicited from a quadrivalent vaccine (Flucelvax) containing both B-V and B-Y HAs. Interestingly, Fluad protected mice from a lethal cross-lineage B-Y viral challenge, while another trivalent vaccine, Fluzone HD, failed to elicit antibodies or full protection following challenge. Fluad immunization also diminished viral burden in the lungs compared to Fluzone and saline groups. The success of a trivalent vaccine to provide protection from a cross-lineage influenza B challenge, similar to a quadrivalent vaccine, suggests that further analysis of different split/subunit vaccine formulations could identify mechanisms for vaccines to target antigenically different viruses. Understanding how to increase the breadth of the immune response following immunization will be needed for universal influenza vaccine development.

## Introduction

Influenza virus has caused global pandemics such as the pandemic of 1918 that caused the death of 50-100 million people (1). Following each of these pandemics there has been an increased focus on the development of vaccines (2). Currently, the ability to produce a pandemic influenza vaccine capable of eliciting a broadly protective immune response to an emergent strain is dependent on the immunogenicity of the current vaccine platforms. For example, in 2009 when a novel H1N1 virus of swine-origin became established in humans the pandemic response relied upon the standard vaccine technologies that were available such as split/subunit influenza vaccines to formulate the pandemic vaccine. Moreover, in addition to the more well-studied seasonal and pandemic activities of influenza type A viruses, the less-studied influenza type B viruses can have epidemic activity (3). Currently, the main seasonal and pandemic responses to influenza use commercial influenza vaccines based on split/subunit vaccines containing influenza hemagglutinin (HA) as the major antigen.

Vaccines to the seasonally circulating strains of influenza have been available for more than 50 years in the United States (4). It is important to study these commercially available vaccines to maximize how they might be utilized and adjusted to improve effectiveness against future pandemic or endemic viruses, like influenza B, since the approval of new vaccine platforms involves a lengthy regulatory process. In the United States, the regulation of influenza vaccines lies with two agencies: the U.S, Food and Drug Administration (FDA) who regulates production and approval of new delivery platforms; and the World Health Organization (WHO) that preforms surveillance of circulating strains and issues semi-yearly recommendations for the strains appropriate for the vaccine. Even when vaccine formulations are well matched to circulating influenza strains, commercial vaccines can sometimes have only at best 60% efficaciousness (5).

In current commercial vaccines HA is the major component and is immunodominant (6), meaning the majority of antibodies target HA. HA targeted antibodies can neutralize virus and protect from influenza infection. Thus, to date HA is the only regulated component in influenza vaccines production (7). The WHO guidance for trivalent vaccines is to contain HA from 2 subtypes of influenza A, representing a H1 and a H3 HA, and a HA corresponding to one lineage of influenza B, representing either the Victoria (B-V) or Yamagata (B-Y) lineage (4). The general structure of HA is conserved among influenza A and influenza B viruses (8, 9). HA is a trimer with each HA0 monomeric protomer cleaved into HA1 and HA2 polypeptides. HA1 is referred to as the head region with mostly beta-sheet structure and contains the receptor binding site while HA2 is referred to as the stem or stalk region with alpha-helical structure and mediates viral membrane fusion. The HA2 stem region is more conserved than that of the HA1 region and numerous strategies have been purposed to increase the HA2 antibodies elicited following vaccination. Some strategies involve increased presentation of the stem region either through engineered nanoparticle displays (10, 11) or *via* chimeric HA molecules (12).

The effectiveness of seasonal influenza vaccines varies from season to season. Seasons with particularly low levels of vaccine success have been attributed to a mismatch between the predicted strain and the circulating strain, as evident in the 2014/5 influenza season where 67% of the infections to H3 were caused by a strain antigenically drifted from the vaccine strain (13). For trivalent vaccines containing only one lineage of influenza B, the selected lineage for vaccine inclusion did not match the circulating lineage 5 times in a 10-year span beginning with the 2001-2 season (14). Most notably, the 2006-7 season recorded 95% of the circulating influenza virus was from the mismatched lineage (15, 16). Beginning in 2012, the FDA approved quadrivalent vaccine formulations which include HA for both predicted lineages of influenza B (17); however, the additional influenza B strain in the commercial vaccines is not required.

The development of new vaccine platforms has focused upon HA epitopes from influenza A viruses due to their pandemic potential. However, influenza B has been estimated as accounting for 1 in 4 influenza infections (18). Influenza B was recognized as 2 distinct lineages, B-Y and B-V, starting from 1983 (19). B-V were originally most prevalent, but in recent years B-V and B-Y have alternated dominance (14). Importantly, strains from both lineages can cause severe disease equal to that of strains of influenza A (20, 21). Influenza B has even been found to have a higher mortality than influenza A in children in some studies (22, 23) and neuraminidase inhibitors, oseltamivir or zanamivir, are less efficacious for treatment of influenza B (24, 25). Human monoclonals antibodies have been isolated that are effective against both lineages of influenza B virus (26, 27). These antibodies were isolated using HA probes and cell sorting techniques and bind to different regions of influenza B hemagglutinin such as the HA1 head and HA2 stem regions (26, 27). Despite the isolation of cross-reactive antibodies to influenza B virus lineages, administration of commercial quadrivalent vaccines with two influenza B lineages remains the currently approved method for eliciting immunity to both influenza B viral lineages in humans (14). However, whether different commercial influenza vaccine formulations induce differences in cross-reactive immunogenicity in sera in terms of influenza B viruses has not been explored in detail as most influenza vaccine studies have focused primarily on influenza type A viruses.

In this study, we selected vaccines that allowed the testing of different formulation parameters on outcomes such as immunogenicity, influenza B lineage cross-reactivity, and protection from heterologous intranasal influenza B viral challenge. One vaccine formulation parameter was whether vaccines contained HAs from the two different influenza B lineages (i.e. quadrivalent, H1, H3, B-V, B-Y) or only HA from one influenza B lineage (i.e. trivalent, H1, H3, B-V),. Our selected commercial vaccines were the quadrivalent Flucelvax (Q) and trivalent Fluzone (T). Another vaccine formulation parameter that was tested was the inclusion of an adjuvant. In this case the commercial vaccine was trivalent vaccine Fluad (T) (H1, H3, B-V) that contains MF59C.1 adjuvant, a squalene oil-in water emulsion. We found by comparison that the adjuvant-containing vaccine (Fluad) appears to improve immunogenicity and outcome measures, specifically protection from cross-lineage influenza B viral challenge. The adjuvanted vaccine (Fluad (T)) with B-V HA could elicit binding antibodies to not only B-V HA but also cross-reactive antibodies to B-Y HA. In addition, when compared to the non-adjuvanted trivalent Fluzone (T), the trivalent adjuvanted Fluad (T) vaccine provided greater protection in mice against a heterologous intranasal influenza B-Y challenge. Our observed differences between the breadth of the trivalent influenza vaccines’ responses to influenza B viruses highlights the importance of further studies on the immunogenicity of commercial influenza vaccines for possible use in pandemic and epidemic preparedness. In addition, although some vaccines contained HA antigens from both influenza B lineages (i.e., quadrivalent), the use of vaccines containing one influenza B lineage HA (i.e., trivalent) provides an appropriate system for identifying and further studying broad immune responses to antigenically divergent lineages of influenza B viruses, which could be useful for universal influenza virus development.

## Materials and methods

### Commercial vaccines

**Figure 1:**
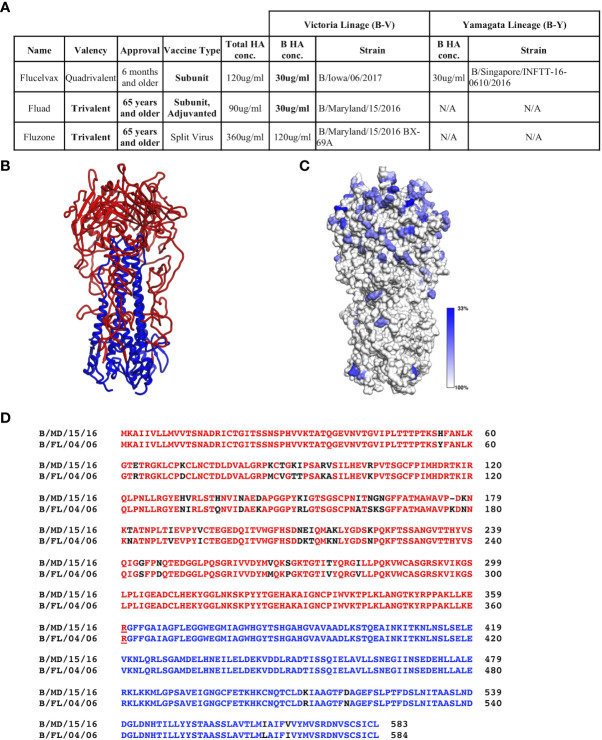
Commercial influenza vaccines used in this study. **(A)** Strain and lineage details for the influenza B HA proteins in the vaccines are provided for the 3 commercial vaccines studied: Flucelvax (Q), Fluad (T) and Fluzone (T). Influenza B HA of Victoria lineage is denoted as B-V, while influenza B HA of Yamagata-lineage is denoted as B-Y. Both the total HA concentration and the concentration of influenza B HA components are indicated. Fluad (T) and Fluzone (T) vaccines are trivalent (H1, H3, influenza B HA of B-V) or quadrivalent like Fluclevax (Q) (H1, H3, B-V, B-Y HAs). H1 and H3 strains are not shown for clarity. **(B)** Structural coordinate model for the trimeric ectodomain for hemagglutinin of influenza B (B/Florida/04/2006, B-Y). HA1 is read with HA2 in blue. **(C)** Conservation of amino acids between influenza B HA proteins in the vaccines and other Flu B HAs used in this study mapped onto a trimeric ectodomain in panel **(B)** Grey is more conserved while blue more variable. **(D)** Example of sequence alignment between a influenza B-V HA (B/Maryland/15/2016) and B-Y HA (B/Florida/04/2006). Colors represent the head (red) and stem (blue) regions of HA, residue differences are shown in black. N/A means not applicable.

Commercial vaccines available for the 2018-9 season were obtained from their manufactures: Sanofi Pasteur (Fluzone (T)) and Seqirus, Inc. (Flucelvax (Q) and Fluad (T)). These vaccines represent the traditional egg adaptive production method along with the more modern approach of production in MDCK cells. The vaccines vary in HA concentration, the presence or absence of a B-Y lineage and the authorized age range for use (**Figure 1A**).

### Mice immunizations

All mouse experiments were performed under protocols approved by the Animal Care and Use Committee (ACUC) at the National Institute of Allergy and Infectious Diseases (NIAID). Female mice were used for all experiments based upon their reported higher immunogenetic response following vaccination (28–30). Initial immunogenicity experiments used 20 BALB/c mice (Taconic Biosciences) aged 8-10 weeks that were randomly assigned to a vaccine group, 5 per group. For immunizations (Prime Day 0 and Boost Day 21), mice were injected intramuscularly (50μL/leg) at the manufacturers supplied concentration, 30 μg/ml per HA (Flucelvax (Q) and Fluad (T)), 120 μg/ml per HA (Fluzone (T)) or saline. Mice were weighed weekly to ensure health with bleeds occurring on Day 0, Day 14, and Day 35 to track immunogenicity.

Mice used for the protection studies were anesthetized and intranasal challenged with 25μl of influenza B/Florida/04/2006 (B-Y) at 10xMLD_50_ (50% Mouse Lethal Dose) on Day 42. This influenza B virus has been previously validated as a mouse challenge model (12). The initial survival experiments used ten mice per vaccine group. Mice were weighed daily and observed twice daily for survival criteria (animals were euthanized if they lost >30% of their initial body weight) until Day 56, when all surviving mice were euthanized. Differences in survival rates were compared using a Kaplan-Meier survival analysis and comparison of survival curves by Log-rank (Mantel-Cox) test.

A replicate mouse study was conducted for organ collection on Day 45, 3 days post-challenge. 20 mice, 5 per group, were assigned to either one of the trivalent vaccine groups, saline control, or a sham inoculation group. The quadrivalent vaccine was not used in this experiment, replaced by the sham inoculation group that received intranasal administration of saline supplemented with 0.1% bovine serum albumin (BSS-BSA) on Day 42 instead of virus challenge.

### ELISA

Immunogenicity of the vaccines was determined by enzyme-linked immunosorbent assay (ELISA) of sera samples collected on Day 35. HA1 and HA0 influenza B HA recombinant antigens were applied to 96-well plates and incubated overnight at 4°C (1.25 μg/mL). Plates were washed (405 TS ELISA Plate Washer, Agilent Technologies) with PBS plus 0.1% Tween 20 between all steps. After blocking (1% Omniblok, AmericanBio, Inc., and 0.1% Tween 20 in PBS), serum samples from vaccinated mice were plated at a 1:125 dilution followed by two-fold dilutions. Plates were then incubated at 37°C with a HRP conjugated secondary antibody (goat anti-mouse IgG (H+L), Thermo Fisher Scientific). Colorimetric detection occurred at room temperature and measured for absorbance at 405 nm (1-Step ABTS, Thermo Fisher Scientific). Endpoint titers levels were statistical defined per plate (31) and analysis of variance (ANOVA) tests were conducted with Tukey’s multiple comparisons test to determine group differences.

### Reporter-based microneutralization assay

Influenza B reporter viruses were prepared similarly to those described previously (32). Briefly, both B-V and B-Y viruses were made with a modified PB1 segment expressing the fluorescent TdKatushka reporter gene (R3ΔPB1) and propagated in MDCK-SIAT-PB1 cells. Replication-restricted reporter influenza viruses encoding influenza B HA and NA coding regions were rescued using plasmids expressing the open reading frames of influenza B HA and NA genes flanked by genome packaging signals of influenza B HA (33) and NA segments (34), respectively. Rescued viruses were propagated in MDCK-SIAT1-PB1 cells in the presence of TPCK-trypsin (1 μg mL-1, Sigma) at 34°C. Virus stocks were stored at -80°C. Mouse sera were treated with receptor destroying enzyme (RDE II; Denka Seiken) and heat-inactivated before use in neutralization assays. Immune sera were serially diluted and incubated for 1 h at 37°C with pre-titrated viruses (B/Phuket/3073/2013 (B-Y), B/Colorado/06/2017 (B-V)). Serum-virus mixtures were then transferred to 96-well plates (PerkinElmer), and 1.0×104 MDCK-SIAT1-PB1 cells (32, 35) were added into each well. After overnight incubation at 37°C, the number of fluorescent cells in each well was counted automatically using a Celigo image cytometer (Nexcelom Biosciences).

### Determination of median tissue culture infectious dose (TCID50)

The concentration of infectious virus in the lungs (left lobe) and nasal turbinate was determined by TCID50 assay in Madin-Darby Canine Kidney (MDCK) cells. Briefly, frozen organs kept at ≤ -65°C were thawed at 37°C ± 2°C for no more than 5 minutes. Once thawed, the organs were homogenized in Dulbecco’s Modified Eagle Medium (DMEM, 10% w/v) (Gibco) using a Bead Ruptor 12 (Omni International) in tubes containing 1.4 mm beads. Homogenized organs were then centrifuged at 2000 x g for 5 minutes to remove cellular debris. The resulting supernatant was diluted 10-fold. After 10-fold serial dilutions were made, 100μL was transferred into respective wells of a 96-well plate which contained a monolayer of MDCK cells. Plates were incubated at 37°C ± 2°C in 5% ± 2% CO2. After 72 hours, the wells were observed for cytopathogenic effect (CPE). TCID50 titers were calculated using the Reed-Meunch method. Differences were assessed *via* ANOVA and Tukey’s multiple comparison test.

### Histology and immunohistochemistry

Formalin-fixed lung for histologic examination were trimmed, processed, and embedded in paraffin according to established protocols. Histology sections were cut at 5µm, mounted on glass slides, and stained with hematoxylin and eosin (H&E). Additionally, serial sections of lung were labeled for immunohistochemical staining using a rabbit polyclonal anti-Influenza B virus nucleoprotein antibody (GeneTex CAT# GTX135819). Staining was carried out on the Bond RX (Leica Biosystems) platform according to manufacturer-supplied protocols. Briefly, sections were deparaffinized and rehydrated. Heat-induced epitope retrieval (HIER) was performed using Epitope Retrieval Solution 1, pH 6.0, heated to 100°C for 20 minutes. The specimen was then incubated with hydrogen peroxide to quench endogenous peroxidase activity prior to applying the primary antibody at a dilution of 1:1000. Detection with DAB chromogen was completed using the Bond Polymer Refine Detection kit (Leica Biosystems CAT# DS9800). Slides were finally cleared through gradient alcohol and xylene washes prior to mounting and placement of coverslips. Sections were examined by light microscopy using an Olympus BX51 microscope and photomicrographs were taken using an Olympus DP73 camera. Scoring of the lung slices for histology and immunohistochemistry was conducted blindly on slides representing all lung lobes utilizing a modified rubric (**Figure S5**) consistent with the field (36, 37). Differences were assessed *via* ANOVA and Tukey’s multiple comparison test.

### HA modeling and sequence alignments

HA sequences were obtained from NCBI (https://www.ncbi.nlm.nih.gov/). A homology model of HA from B/Florida/04/2006 was created using SWISS-MODEL server (38), which identified structural template 2WRF.3.A (H2) with a global model quality estimate (GMQE) of 0.51, a quaternary structure quality estimate (QSQE) of 0.60, and a sequence identity of 28.5% for threading and modeling with the B/Florida/04/2006 HA sequence. The resulting model had a QMEANDisCo Global of 0.53±0.05. To visualize sequence identities, multiple sequence alignments were preformed using MUSCLE (39), and mapped onto the structure using UCSF Chimera (40).

## Results

### Conservation of influenza B hemagglutinins sequences

While it is known that influenza B HAs have conserved regions, before assessing vaccine immunogenicity we used bioinformatics to assess the sequence conservation between influenza B HAs used in this study. Flu B HA sequences included those in the commercial influenza vaccines, recombinant influenza B HA proteins, and influenza B reporter viruses (**Figure 1A**, **Figure S1**). We did this to confirm that there were conserved regions among the influenza B HAs even though they are from different influenza B lineages and strains. All influenza B HAs had greater then 90% sequence identity between pairwise comparisons (**Figure S2**). Although the trimeric ectodomain structure is conserved among influenza B HAs there are residues that show variation with move variation in the HA1 head region (**Figures 1B, C**). A closer inspection of the primary sequences of a B-V and B-Y lineage HAs (HA0) used in this study indicated a 92.97% sequence identity (**Figure 1D**). However, when comparing HA1 and HA2 sequences separately the HA1 sequence identity decreases to 89.5% while the HA2 sequence identity increases to 98.2%. Thus, there are less sequence changes in the HA2 sequence than HA1 sequence between the two lineages (**Figure 1B**, black residues). However, despite B-V and B-Y HA antigens having higher sequence identity than H1 versus H3 HAs (e.g., 42.4%), quadrivalent vaccines are still formulated to have the two different lineages of influenza B HA. This is done to elicit protective immune responses to both influenza B lineages. Thus, we wanted to probe for possible differences in the elicitation of cross-reactive (i.e., cross-lineage) influenza B HA antibody responses between different commercial influenza vaccines that can vary in their manufacturing procedures such as chemicals and detergents used for HA antigen purification. Also, because HA1 showed more antigenic variation than HA2 we used both HA1 heads and HA0 trimeric proteins to assess the elicited cross-reactivity of vaccines.

### Differences in the elicitation of cross-reactive antibodies to influenza B HA proteins

**Figure 2:**
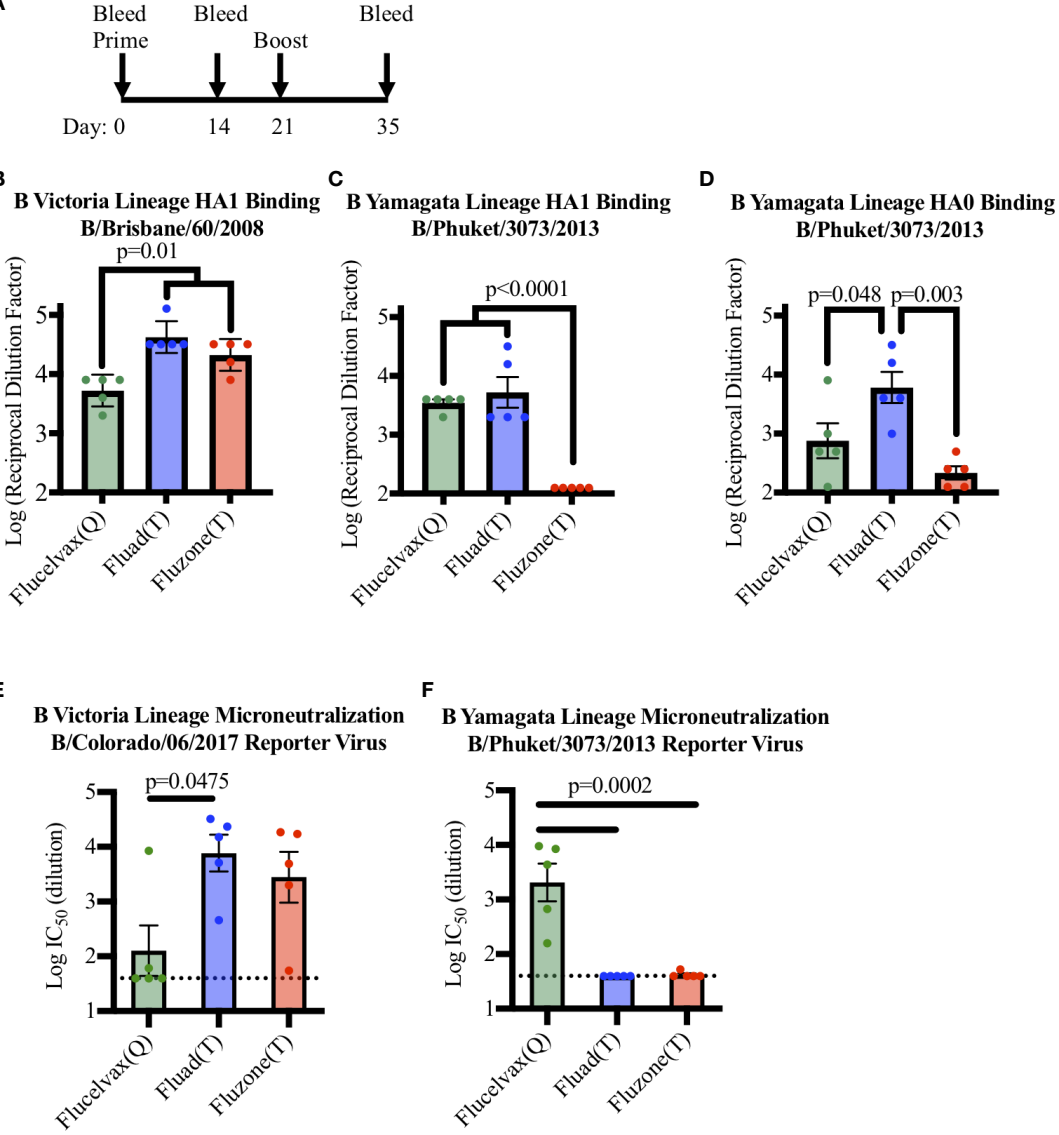
Comparing antibody and microneutralization levels of vaccine sera against influenza B Victoria and influenza B Yamagata lineages: **(A)** Immunization schedule indicating days for the prime and boost for the vaccines (day 0 and 21) and bleeds (day 0, 14, and 35). **(B–D)** ELISA binding of immunized mouse sera to influenza B virus recombinant HA proteins: **(B)** HA1 of B-V (B/Brisbane/60/08), **(C)** HA1 of B-Y (B/Phuket/3073/13), **(D)** HA0 of B-Y (B/Phuket/3073/13), P-values are indicated for the significance of differences. **(E, F)** Testing microneutralization activities of vaccine sera using influenza B reporter viruses derived from **(E)** B-V (B/Colorado/06/2017) and **(F)** B-Y (B/Phuket/3073/2013). Vaccines were Flucelvax (Q) (green), Fluad (T) (blue) and Fluzone (T) (red). The dotted horizontal lines in panels E and F indicates the threshold for detection above background in the assay.

To determine if a set of commercial influenza vaccines showed differences in immunogenicity in terms of eliciting antibodies to HA from the two lineages of influenza B virus, mice were immunized with vaccines in a prime boost administration regimen (**Figure 2A**) and cross-reactivity was probed by ELISA (**Figure S3**). All the vaccines contained influenza B HA from B-V and all immunized mice were able to produce antibodies capable of binding to recombinant HA1 protein from B-V in ELISA (**Figure 2B**). However, even though all vaccines contained B-V HA, statistical analysis indicated that vaccine sera differed in their levels of B-V HA1 binding antibodies (**Figure 2B**, F (2, 12)=14.58, p=0.006). When the groups were further compared, the concentration of HA in the vaccine corelated with immunogenicity, with the higher antigen dose of Fluzone (T) (12ug of HA B-V per injection) producing a higher immune response compared to Flucelvax (Q) (p=0.011) (**Figure 2B**, red v. green bars). However, the addition of an adjuvant removed this antigen concentration effect as Fluad (T) and Flucelvax (Q) both delivered 3μg of HA B-V per injection, but Fluad (T) elicited more antibodies than Flucelvax (Q) (p=0.005) (**Figure 2B**, blue vs. green bars) and showed binding equal to Fluzone (T) (p=0.222) (**Figure 2B**, blue and red bars).

Interestingly, the trivalent adjuvanted vaccine Fluad (T) (without B-Y HA) elicited levels of cross-reactive antibodies to HA1 of B-Y that were comparable to the quadrivalent vaccine Flucelvax (Q) (with B-Y HA) (**Figure 2C**, green and blue bars). For example, when mouse sera were analyzed for binding to a recombinant HA1 protein from B-Y, which is only a component of the quadrivalent influenza formulation, there were differences between the groups in HA1 B-V binding (F (2, 12)=32.85, p<0.001). Follow up analysis showed the quadrivalent vaccine, Flucelvax (Q), was no different than the adjuvanted vaccine, Fluad (T) (p=0.697) but both produced more antibodies than Fluzone (T) (p<0.0001) (**Figure 2C**, green, blue, red bars). However, when a full-length HA0 B-Y protein was used to compare the cross-reactivity of the elicited antibodies the Fluad (T) vaccine without B-Y HA produced the higher levels of reactive antibodies (F (2, 12)=9.484, p=0.003) (**Figure 2D**, blue bar). The Fluad (T) adjuvanted vaccines had higher levels of cross-reactive antibodies than both the quadrivalent vaccine Flucelvax (Q) (p=0.048) and the trivalent vaccine high-dose Fluzone (T) (p<0.003) (**Figure 2D**, blue bar vs. green, red bars).

### Differences of *in vitro* neutralization and *in vivo* protection from viral challenge

To assess the neutralization ability of commercial influenza vaccines against influenza B viruses from both B-V and B-Y lineages, sera from vaccinated mice were used in an influenza B reporter-based microneutralization assay (**Figure S4**). For the homologous influenza B-V reporter virus all the three vaccine sera displayed neutralization activity above threshold (**Figure 2E**). While all vaccines contained B-V HA antigens the level of neutralization from Flucelvax (Q) sera was less than that of Fluad (T) (p=0.0475) and Fluzone (T) (**Figure 2E**, green vs blue bar.). However, only the B-Y HA containing Flucelvax (Q) displayed neutralization above threshold when a B-Y reporter virus was utilized (**Figure 2F**, green bar). The sera from mice immunized with adjuvanted Fluad (T) and Fluzone (T) were not above the established threshold for the assay, suggesting that cross-reactive B-Y HA binding antibodies elicited by Fluad (T) (**Figures 2C, D**, blue bars) were non-neutralizing (**Figure 2F**, blue bar). Thus, because the adjuvanted Fluad (T) sera showed *via* ELISA cross-reactive antibody binding to influenza B-Y HA protein not formulated in the Fluad (T) vaccine (**Figures 2C, D**, blue bars) but Fluad (T) sera did not reach the threshold for B-Y neutralization (**Figure 2F**, blue bar), we further characterized and compared the ability of the commercial vaccines to elicit protection using a influenza B-Y challenge model in mice.

**Figure 3:**
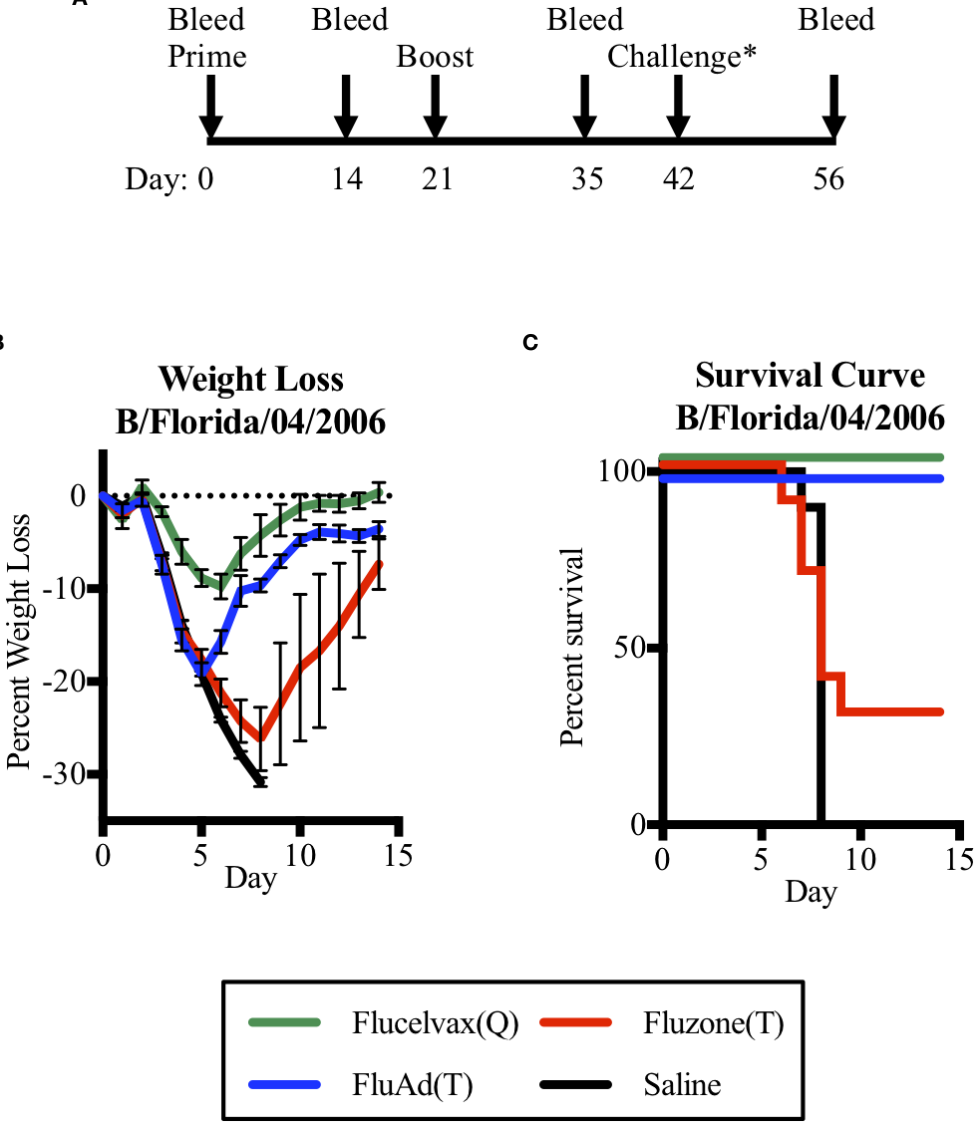
Assessing mice survival and weight loss between quadrivalent (Q) and trivalent (T) vaccines following influenza B (Yamagata-lineage) challenge. **(A)** Immunization and challenge schedule with prime and boost (day 0 and 21), intranasal challenge (day 42), and bleeds (day 0, 14, 35, and 56). **(B)** Weight-lost curves for 14 days following intranasal influenza B-Y challenge. **(C)** Corresponding survival curves. Vaccines were Flucelvax (Q) (green), Fluad (T) (blue) and Fluzone (T) (red) with saline control (black). Yamagata-lineage challenge virus was mouse-adapted B/Florida/04/2006. Challenge* denotes on the timeline when the challenge virus of Mouse Adapted B/Florida/04/2006 (Yamagata Lineage) was administered intranasally.

Thus, to address the question of could a trivalent vaccine with only one influenza B lineage HA protect against a heterologous challenge of the other B lineage, we vaccinated mice and then compared their survival in a challenge model. We found that the adjuvant Fluad (T) formulated without B-Y HA could protect against a heterologous challenge with mouse-adapted B-Y virus (B/Florida/04/2006) (**Figure 3**). To assess if vaccination was protective, mice underwent the same prime boost schedule as the previous immunogenicity experiment with a viral challenge occurring on day 42. Mice were then observed for an additional 14 days to assess outcomes (**Figure 3A**). Mice were weighed daily with an overall trend for virus-induced weight loss beginning 3 days post-challenge and weight gain beginning on day 6 for those that recovered (**Figure 3B**). Mice immunized with the quadrivalent vaccine Flucelvax (Q) (**Figure 3B**, green line) lost less than 10% of their starting weigh, while those immunized with the trivalent vaccines, Fluad (T) or Fluzone (T), and saline control mice lost more than 15% of their starting weight or more (**Figure 3B**, blue, red, black lines, respectively). Those mice that lost 30% of their initial body weight were euthanized as an experimental endpoint (**Figure 3C**).

There were differences in survival based upon immunization group (p<0.0001). Mice immunized with trivalent Fluzone (T) showed a modest level of protection with 30% of mice surviving; however, this was not statistically better than immunization with saline (p=0.369). When compared to the other trivalent vaccine, Fluad (T) immunization was able to significantly improve survival outcome (p=0.002), despite the majority of its antibodies being non-neutralizing against a B-Y reporter-virus in cell culture (**Figure 2F**). The influenza B-Y challenge represented a heterologous challenge for Fluad (T) and Fluzone (T) vaccinated mice and a homologous challenge for Flucelvax (Q) vaccinated mice. Interestingly, mice receiving adjuvanted Fluad (T) showed 100% survival protection from the heterologous mouse-adapted Yamagata influenza B challenge which was equal to the protection from a homologous challenge provided by the quadrivalent vaccine Flucelvax (Q) (**Figure 3C**, green vs blue lines).

### Amount of virus in nasal turbinates and lung tissues and lung pathology

**Figure 4:**
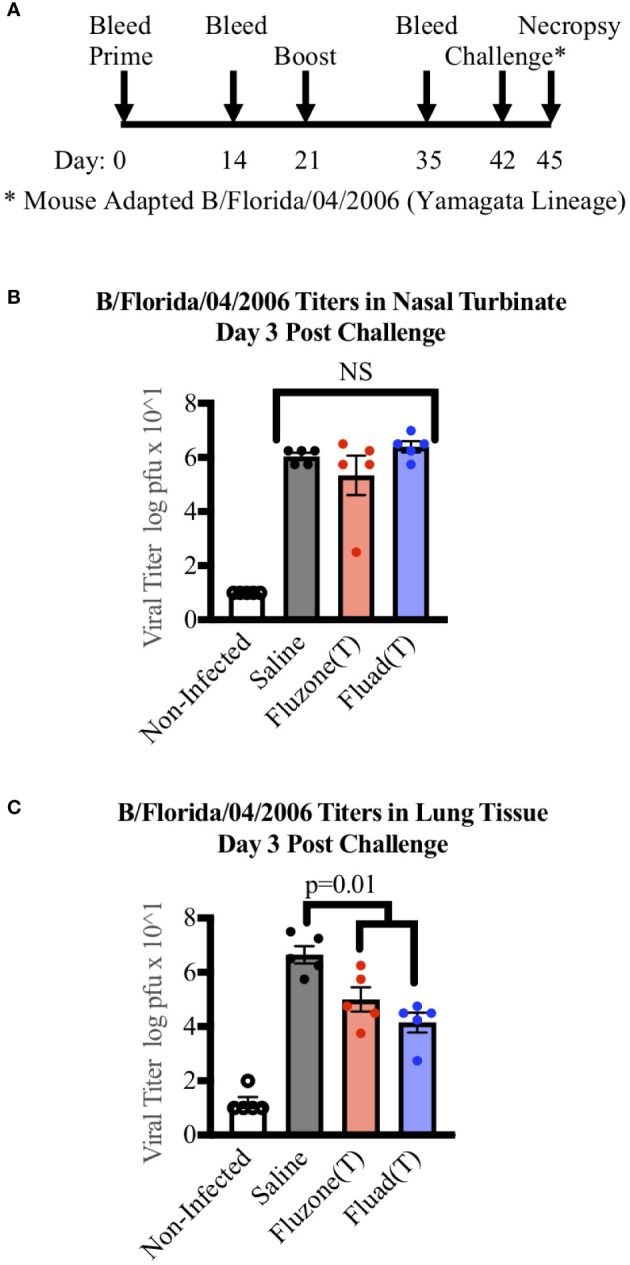
Measuring viral titers in the nasal turbinates and lungs of mice following vaccinations and subsequent challenge with Yamagata-lineage influenza B virus. **(A)** Immunization and challenge schedule with prime and boost (day 0 and 21), intranasal challenge (day 42), and bleeds (day 0, 14, and 35). On day 45 mice were euthanized for the harvesting of lungs and nasal turbinates. **(B)** Viral levels in the nasal turbinates of mice as measured by tissue culture infectious dose 50 (TCID50). **(C)** Viral levels in the left lung of mice as measured by TCID50. P-values are indicated for the significance of differences and NS denotes no statistical difference. Vaccines were Fluad (T) (blue) and Fluzone (T) (red) with saline (black) and non-infected controls (white). Yamagata-lineage challenge virus was mouse-adapted B/Florida/04/2006.

To examine if there were differences in virus clearance following challenge, mice were immunized with one of the trivalent vaccines (Fluad (T) or Fluzone (T)) or saline and intranasally challenged with a mouse-adapted Yamagata lineage influenza B virus (B/Florida/04/2006). But rather than being observed for survival criteria, mice were euthanized three days post-challenge for organ harvesting (**Figure 4A**). The nasal turbinates and left lung samples from challenged mice showed evidence of viral replication (**Figures 4B, C**). Virus levels in the nasal turbinates were not statistically different among the vaccinated groups and saline (**Figure 4B**). In contrast, the saline group had higher virus titers detected in lung samples when compared to mice vaccinated with Fluad (T) (p<0.001) or Fluzone (T) (p=0.018) (**Figure 4C**). There was not a significant difference in virus lung titers detected between the Fluad and Fluzone vaccinated groups (**Figure 4C**). These vaccines are not considered epithelial protectant and therefore it’s not unexpected to see changes to the epithelium of large airways due to influenza B infection.

**Figure 5:**
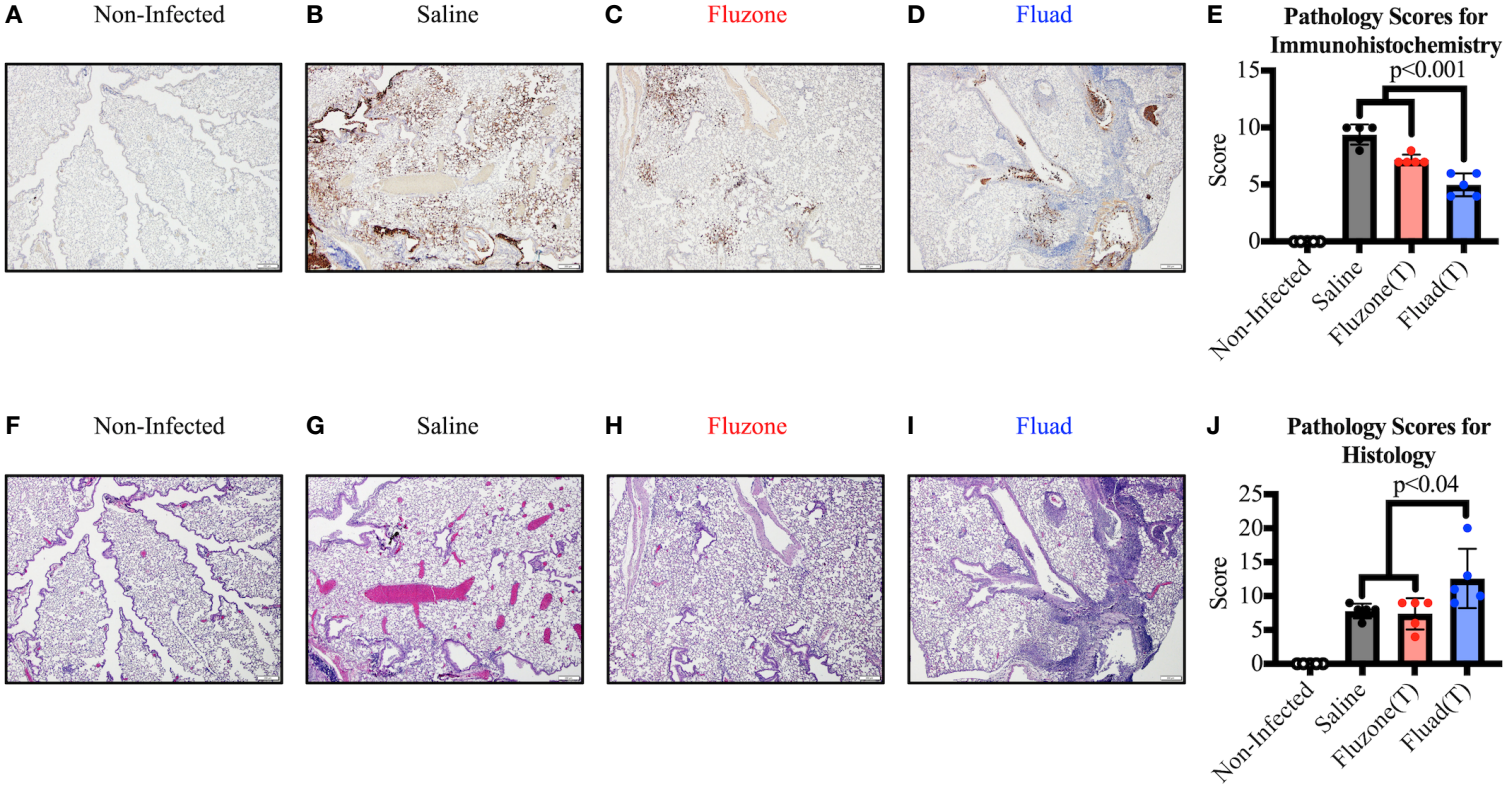
Immunohistochemistry and histopathology of mouse lungs following vaccination with commercial influenza vaccines and subsequent challenge with Yamagata lineage influenza B virus. **(A–D)** Representative images show immunohistochemistry (IHC) against the influenza B nucleoprotein protein for different vaccination groups. **(A)** Uninfected lungs from sham inoculated mice exhibit no viral antigen staining. **(B)** Positive-control lung from the saline vaccinated group displays viral antigen (brown) along large airway epithelium and affecting greater than 50% of the alveolar interstitium. **(C)** Lung from the Fluzone (T) vaccinated group shows considerably less viral antigen (brown) in the alveolar interstitium compared to the positive control. **(D)** Lung from the Fluad (T) vaccinated group displays viral antigen (brown) in less than 10% of the interstitium; the majority of the antigen present is associated with airway epithelium and within airway lumina. All images at 4x, (scale bar is 200μm). **(E)** Immunohistochemical scoring based on relative amount of influenza antigen in sections. **(F–I)** Serial sections of lung were used to detect inflammation by routine H&E. **(F)** Negative-control lung from non-infected group is histologically normal. **(G)** Positive-control lung from the saline vaccinated group displays airway epithelial changes but minimal changes in the alveolar interstitium. **(H)** Lung from the Fluzone (T) vaccinated group exhibit similar airway changes as the positive-control lung. **(I)** Lung from the Fluad (T) vaccinated group shows similar pulmonary changes as well as robust perivascular inflammation in some areas. All images at 4x, (scale bar is 200μm). **(J)** Histology scoring based on inflammation in sections.

However, when sections of the right lung were stained and scored for virus antigen (**Figures 5A–E**), there was decreased viral antigen detected in the alveolar interstitium of animals vaccinated with Fluad (T) or Fluzone (T) compared with saline (p<0.001). Fluad (T) immunized mice had significantly less viral antigen staining than Fluzone (T) immunized mice (p<0.001) (**Figure 5E**). However, when comparing histologies of the lung groups (**Figures 5F–J**) the adjuvanted Fluad (T) lungs showed greater levels of inflammation particularly in the perivascular areas (**Figure 5I**), as measured by histology scores, compared to mice vaccinated with Fluzone (T) (p=0.02) or Saline controls (p=0.04) (**Figure 5J**). Saline and Fluzone (T) groups showed no difference in lung inflammation following challenge. Non-infected control lungs were histologically normal with no evidence of viral antigen staining (**Figures 5F–J**). The greater levels of inflammation of Fluad (T) as observed by histology (**Figures 5I, J**) is consistent with the reports that adjuvant MF59 induces inflammatory cytokines (41).

## Discussion

Predicting future strains of circulating influenza virus is difficult, and mismatch with vaccine strains is inevitable with the current technologies. To improve outcome measures following vaccination, commercially available influenza vaccines should be formulated to elicit broadly cross-reactive antibodies. Trivalent vaccines only have one lineage of influenza B and offer the unique ability to test for cross-reactivity to the circulating strain from the other lineage, modeling human exposure (42). In human populations, Camilloni et al. showed that immunization with an adjuvanted trivalent vaccine produced HAI titers to both lineages of influenza B. However, the human participants had previous natural exposure to both circulating lineages and many participants had cross-reactive antibodies prior to experimentation (43). In this experiment, we utilized naïve mice to determine if the cross lineage immune response is attributed to vaccination. We compared three commercial vaccines, two trivalent and one quadrivalent, for their ability to elicit antibodies to both lineages of influenza B and if those antibodies could protect from a heterologous challenge.

From our work, sequence comparisons of the influenza B HA antigens formulated in the vaccines had regions that were conserved. These regions indicate the presence of conserved epitopes for the possible elicitation of cross-reactive antibodies between the Victoria and Yamagata lineages of influenza B viruses (**Figure 1**). These conserved influenza B HA epitopes might be useful in universal influenza vaccine development. The development of universal influenza vaccines is a major goal of the biomedical research community (44). Novel approaches and technologies to vaccine development include immunogen designs, gene-based vaccine platforms and formulations of recombinant antigens with potent adjuvants (45). However, how parameters such as antigen formulation and adjuvant addition might affect outcomes such as influenza B cross-lineage immunogenicity, protection, and pathology have not been explored in detail when it comes to currently approved commercial influenza vaccines.

In this study all of the vaccines tested were immunogenetic for the homologous influenza B Victoria Lineage (B-V). The trivalent vaccines were able to elicit a significantly higher antibody level for the homologous influenza B-V HA1 protein compared to the quadrivalent vaccine (Flucelvax (Q)) (**Figure 2B**); this difference in immunogenicity was not unexpected as both trivalent vaccines are approved for use in an elderly population which require an elevated immune response (**Figure 1A**). The trivalent vaccines have an adjuvant (i.e., Fluad (T)) and a higher HA concentration (i.e., Fluzone (T)) than the quadrivalent vaccine Flucelvax (Q). Unsurprisingly, when the influenza B Yamagata lineage (B-Y) HA1 protein was used to assess binding, the quadrivalent Flucelvax (Q) was able to elicit reactive antibodies and the trivalent Fluzone (T) did not. However, the adjuvanted trivalent vaccine Fluad (T) was able to elicit heterologous cross-reactive antibodies to a similar level as the quadrivalent vaccine, despite not containing the matched virus strain for B-Y (**Figure 2C**). Furthermore, when HA0 trimeric protein of B-Y was used to probe for cross-reactive antibodies the Fluad (T) displayed the highest levels (**Figure 2D**). Thus, in terms of formulation parameters our results suggest that having only just a higher protein concentration alone (i.e., Fluzone (T)) might not be sufficient to elicit cross-lineage antibodies to influenza B but the addition of adjuvant (i.e., Fluad (T)) is important.

While much of vaccine development focuses on neutralizing antibody activity, it is becoming appreciated that non-neutralizing antibodies also can play a role in an effective immune response against viruses such as influenza (46–50). A number of studies have indicated the role of cross-reactive non-neutralizing antibodies in providing cross-protection against antigenically divergent heterologous influenza viral strains (47, 50, 51). In contrast to neutralizing antibodies, cross-reactive non-neutralizing can recognize epitopes that are highly conserved such as the stem region of HA (47, 48, 50, 51). Also, non-neutralizing antibodies have been reported to bind the viral proteins on the surface of infected cells and mediate cross-protection *via* Fc receptor mediated activities (46, 47, 50, 51).

In our study when mouse sera were used in virus neutralization assays, again all vaccines were able to produce antibodies capable of homologous neutralization of a matched B-V virus (**Figure 2E**). However, when a B-Y virus was utilized, only the quadrivalent vaccine Flucelvax had homologous neutralization activity while the trivalent vaccines not containing B-Y antigen did not have heterologous neutralization activity above threshold (**Figure 2F**). Thus, Fluad (T) elicited antibodies could be operationally defined as non-neutralizing. Fluad (T) sera antibodies displayed heterologous binding in ELISA to both recombinant HA1 and trimeric HA0 of B-Y not formulated in the vaccines (**Figures 2C,D**). However, the Fluad (T) sera antibodies neutralized a homologous matched B-V virus (**Figure 2E**) but were non-neutralizing against an unmatched heterologous B-Y virus (**Figure 2F**). Based on the literature, our results suggest that Fluad (T) is eliciting non-neutralizing cross-reactive antibodies to the stem region of B-Y HA (**Figure 2D**). This is consistent with the elicitation of stem antibodies which tend to have very low or below threshold activities in viral neutralization assays. While not yet reported for influenza B, immunization with Fluad (T) has previously been shown to elicit HA stem antibodies for influenza A (52). It will be interesting in future studies to further define the cross-reactive antibody response to the B-Y virus that was elicited by the adjuvanted trivalent Fluad (T) vaccine to determine what epitopes are being recognized. It will be important to access if Fluad (T) is eliciting antibodies to novel conserved influenza B HA epitopes or antibodies to conserved influenza B HA epitopes previously identified using monoclonal antibodies (26, 27, 53).

Although human challenge models for influenza are available (54) it is logistically challenging to correlate cross-protective but non-neutralizing immune responses to actual lung pathology. This is particularly unfeasible/unethical for human studies using commercial influenza vaccines because human viral challenges are at sublethal doses. Therefore, mice provide an advantageous model for protection studies. We proceeded to challenge mice with a mouse-adapted influenza B-Y virus. Immunization with adjuvanted Fluad (T) protected 100% of mice from the lethal heterologous B-Y challenge (**Figure 3**). Conversely, Fluzone (T) immunization was not able to protect mice from virus challenge. At face value this suggests that in our experiments, neither HA binding nor microneutralization assay definitively correlated with protection from heterologous challenge elicited by Fluad (T) and Fluzone (T). Although one caveat of our work is that we did not test for Fc-mediated activity, our results are consistent with Fluad (T) eliciting non-neutralizing but cross-reactive and cross-protective antibodies to influenza B-Y (**Figures 2**, **3**) (47, 48). The implication for influenza B vaccine development is that multiple assays should be used when accessing vaccine immunogenicity, protection, and pathology.

To examine differences in viral pathology a mouse model allows for the assessment of lung tissue usually impossible in human studies. However, there have been relatively few mouse pathology experiments done utilizing influenza B lineage viruses compared to those examining influenza A. One study, utilizing the same B-Y virus as our challenge virus showed similar pathology to our saline group (55). However, that study did not include any groups anticipated to survive the viral challenge. When examining other outcome measures following viral challenge, such as virus staining and inflammation, antibodies elicited from Fluad (T) immunizations offered more of an advantage than those from Fluzone (T). While both immunization groups and the saline control mice had similar levels of virus detected in the nasal turbinates, the vaccinated groups have significantly less virus in their lungs detected by TCID50 (**Figure 4**). When sections of lung are compared, the viral antigen in the lower respiratory tract is more prominent in lungs from mice immunized with Fluzone (T) (<50%) compared to those immunized with Fluad (T) (5-10%) (**Figure 5**). Fluad (T) immunized mice also had increased inflammation scores compared to Fluzone (T) immunized mice. Therefore, to interpret our histological findings we relied upon the literature for influenza A. Following infection with influenza A, apoptosis of monocyte/alveolar macrophage cells trigger an increase in pro-inflammatory cytokines that is associated with migration of B and T cells to the site of infection, overall viral clearance, and improved survival (56, 57). This suggests that the inflammation response following influenza B challenge, which is seen most predominately in lungs of mice immunized with Fluad (T), is a precursor to viral clearance and survival from challenge. Furthermore, our results suggest that the relatively increased inflammatory response observed in the lungs for mice immunized with Fluad (T) could be because of both the reported induction of inflammatory cytokines by the adjuvant MF59 (41) and the use of a mouse adapted influenza B-Y virus (12). The use of a virus that was adapted to be pathogenetic in mice is necessary because in general human influenza B viruses do not display sufficient pathology in mice to test vaccine efficaciousness (58). Interestingly, one clinical study demonstrated that influenza vaccine with the MF59 adjuvant was efficacious against PCR-confirmed influenza in infants and young children (59). One notion from our work is that perhaps in future experiments increasing the influenza B HA concentration of Fluad (T) to that of the high-dose Fluzone (T) might increase the level of cross-reactive antibodies to both influenza B lineages. Also, in future studies it would be interesting to see how different adjuvants might function in increasing the immune response but reducing inflammation.

Mechanistically, our study suggests that the Fluad (T) adjuvanted vaccine protects against a heterologous influenza B viral challenge *via* eliciting HA binding, but non neutralizing antibodies that function similar to other reported non-neutralizing antibodies (47, 48). While the addition of an adjuvant has been previously shown to elicit cross-lineage protection (60), this is the first reported study with appropriate controls to attribute that capability to a commercially available adjuvanted vaccine. The ability of an adjuvanted vaccine to improve survival outcomes following heterologous challenge needs further investigation as to the mechanism of protection, as adjuvants have been hypothesized to improve immunogenicity through multifaceted mechanisms (61, 62). Use of influenza B as a model of cross-reactive antibodies is also critical as there has been speculation that the lineages could diverge again (3), which would further complicate the creation of a universal influenza vaccine. Based on our results from mice and previous MF59 clinical studies (43, 59) one clinical implication of our work is that further clinical studies with the use of MF59 as an adjuvant should be explored. This will aid understanding the extent to which MF59 can elicit cross-protective immune responses to antigenically different influenza viruses. Although numerous platforms such as nanoparticles and whole-inactivated viruses are being explored to develop more broadly protective vaccines against the influenza B viruses from the two currently circulating lineages (B-V and B-Y) (10, 63), our work suggest further studies on how to optimize the elicited cross-protective immune responses of current commercial vaccines might be useful in improving seasonal influenza vaccines. Also, these improved formulations of commercial vaccines could be repurposed for universal influenza vaccines studies.

## Data availability statement

The original contributions presented in the study are included in the article/**Supplementary Material**. Further inquiries can be directed to the corresponding author.

## Ethics statement

The animal study was reviewed and approved by the Animal Care and Use Committee (ACUC) at the National Institute of Allergy and Infectious Diseases (NIAID).

## Author contributions

MM designed the *in vivo* experimental studies and ELISA experiments. DW, RS-C, SM-P, and JG carried out viral sequence alignments and analysis. KB and DA conducted and interpreted the pathology experiments. SB-B, HS, AC, and MK performed the B virus reporter assay and analysis. MM, JG, and AH designed the study and experiments and wrote the paper. All author contributed to editing the paper. All authors read and approved the final manuscript.

## Funding

This work was supported by the Intramural Research Program of the National Institute of Allergy and Infectious Diseases. Intramural award ZIAAI001180.

## Acknowledgments

We thank the International Reagent Resource for HA proteins. We thank Dr. Florian Krammer and IIT Research Institute for the influenza B challenge virus.

## Conflict of interest

The authors declare that the research was conducted in the absence of any commercial or financial relationships that could be construed as a potential conflict of interest.

## Publisher’s note

All claims expressed in this article are solely those of the authors and do not necessarily represent those of their affiliated organizations, or those of the publisher, the editors and the reviewers. Any product that may be evaluated in this article, or claim that may be made by its manufacturer, is not guaranteed or endorsed by the publisher.
