# Corrigendum: Impact of adjuvant: trivalent vaccine with quadrivalent-like protection against heterologous Yamagata-lineage influenza B virus

**DOI:** 10.3389/fimmu.2023.1283355

**Published:** 2023-09-12

**Authors:** Mallory L. Myers, John R. Gallagher, De’Marcus D. Woolfork, Regan K. Stradtmann-Carvalho, Samantha Maldonado-Puga, Kevin W. Bock, Seyhan Boyoglu-Barnum, Hubza Syeda, Adrian Creanga, Derron A. Alves, Masaru Kanekiyo, Audray K. Harris

**Affiliations:** ^1^ Structural Informatics Unit, Laboratory of Infectious Diseases, National Institute of Allergy and Infectious Diseases, National Institutes of Health, Bethesda, MD, United States; ^2^ Infectious Disease Pathogenesis Section, National Institute of Allergy and Infectious Diseases, National Institutes of Health, Bethesda, MD, United States; ^3^ Vaccine Research Center, National Institute of Allergy and Infectious Diseases, National Institutes of Health, Bethesda, MD, United States

**Keywords:** influenza B, MF59 adjuvant, commercial vaccine, challenge, Yamagata lineage

In the published article, there was an error in the legend for **Figure 4** as published. “quadrivalent and trivalent vaccinations” should have been “vaccinations” and “Flucelvax (Q) (green)” removed. The corrected legend appears below.

**Figure 4:**
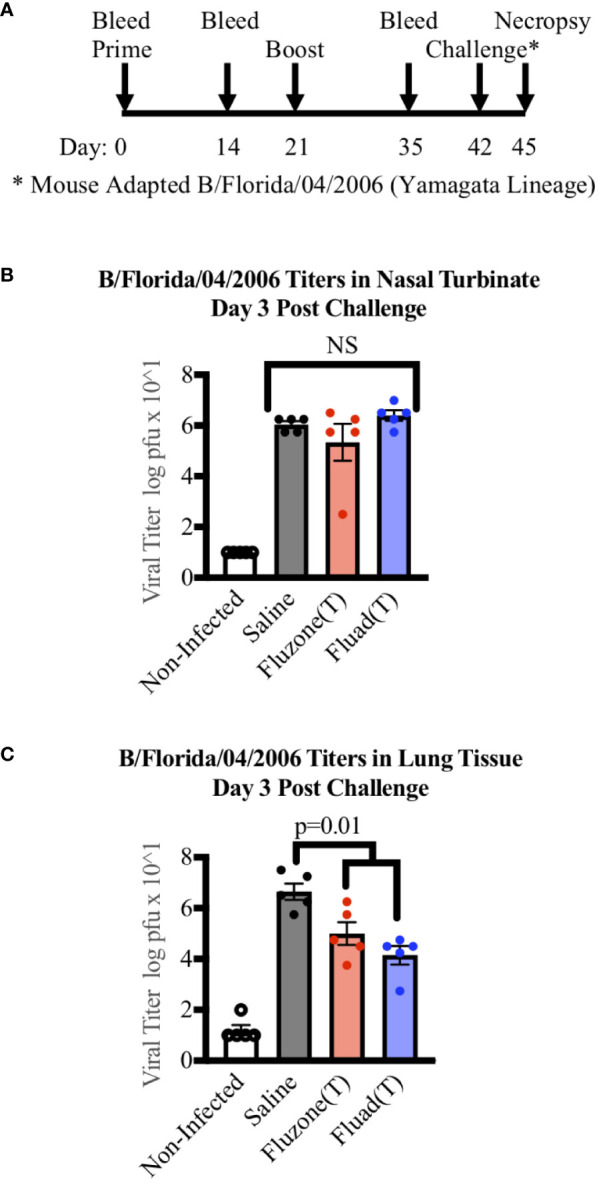
Measuring viral titers in the nasal turbinates and lungs of mice following vaccinations and subsequent challenge with Yamagata-lineage influenza B virus. **(A)** Immunization and challenge schedule with prime and boost (day 0 and 21), intranasal challenge (day 42), and bleeds (day 0, 14, and 35). On day 45 mice were euthanized for the harvesting of lungs and nasal turbinates. **(B)** Viral levels in the nasal turbinates of mice as measured by tissue culture infectious dose 50 (TCID50). **(C)** Viral levels in the left lung of mice as measured by TCID50. P-values are indicated for the significance of differences and NS denotes no statistical difference. Vaccines were Fluad (T) (blue) and Fluzone (T) (red) with saline (black) and non-infected controls (white). Yamagata-lineage challenge virus was mouse-adapted B/Florida/04/2006.

“Measuring viral titers in the nasal turbinates and lungs of mice following vaccinations and subsequent challenge with Yamagata-lineage influenza B virus. **(A)** Immunization and challenge schedule with prime and boost (day 0 and 21), intranasal challenge (day 42), and bleeds (day 0, 14, and 35). On day 45 mice were euthanized for the harvesting of lungs and nasal turbinates. **(B)** Viral levels in the nasal turbinates of mice as measured by tissue culture infectious dose 50 (TCID50). **(C)** Viral levels in the left lung of mice as measured by TCID50. P-values are indicated for the significance of differences and NS denotes no statistical difference. Vaccines were Fluad (T) (blue) and Fluzone (T) (red) with saline (black) and non-infected controls (white). Yamagata-lineage challenge virus was mouse-adapted B/Florida/04/2006.”

The authors apologize for this error and state that this does not change the scientific conclusions of the article in any way. The original article has been updated.

