# Supplementary material for: Impact of adjuvant: Trivalent vaccine with quadrivalent-like protection against heterologous Yamagata-lineage influenza B virus
Source: Front Immunol. 2022 Sep 30;13:1002286. doi: 10.3389/fimmu.2022.1002286 (PMC9561127; doi:10.3389/fimmu.2022.1002286)
Supplement: Supplementary file 1 [file DataSheet_1.pdf]

Supplementary Material

Supplementary Figures

Victoria Lineage (B-V)

Use	Strain	Sequence
Vaccine	B/Maryland/15/2016	MKAIIVLLMVVTSNADRICTGITSSNSPHVVKTATQGEVNVTGVIPLTTTPTKSHFANLKGTETRGKLCP<br>KCLNCTDLDVALGRPKCTGKIPSARVSILHEVRPVTSGCFPIMHDRTKIRQLPNLLRGYEHVRLSTHNVI<br>NAEDAPGGPYKIGTSGSCPNITNGNGFFATMAWAVPDKNKTATNPLTIEVPYVCTEGEDQITVWGFHSDN<br>EIQMAKLYGDSKPQKFTSSANGVTTHYVSQIGGFPNQTEDGGLPQSGRIVVDYMVQKSGKTGTITYQRGI<br>LLPQKVWCASGRSKVIKGSLPLIGEADCLHEKYGGLNKSKPYYTGEHAKAIGNCPIWVKTPLKLANGTKY<br>RPPAKLLKERGFFGAIAGFLEGGWEGMIAGWHGYTSHGAHGVAVAADLKSTQEAINKITKNLNSLSELEV<br>KNLQRLSGAMDELHNEILELDEKVDDLRADTISSQIELAVLLSNEGIINSEDEHLLALERKLKKMLGPSA<br>VEIGNGCFETKHKCNQTCLDKIAAGTFDAGEFSLPTFDSLNITAASLNDDGLDNHTILLYYSTAASSLAV<br>TLMIAIFVVYMVSRDNVSCSICL
	B/Iowa/06/2017	MKAIIVLLMVVTSNADRICTGITSSNSPHVVKTATQGEVNVTGVIPLTTTPTKSHFANLKGTETRGKLCP<br>KCLNCTDLDVALGRPKCTGKIPSARVSILHEVRPVTSGCFPIMHDRTKIRQLPNLLRGYEHVRLSTHNVI<br>NAEGAPGGPYKIGTSGSCPNITNGNGFFATMAWAVPDKNKTATNPLTIEVPYVCTEGEDQITVWGFHSDN<br>ETQMAKLYGDSKPQKFTSSANGVTTHYVSQIGGFPNQTEDGGLPQSGRIVVDYMVQKSGKTGTITYQRGI<br>LLPQKVWCASGRSKVIKGSLPLIGEADCLHEKYGGLNKSKPYYTGEHAKAIGNCPIWVKTPLKLANGTKY<br>RPPAKLLKERGFFGAIAGFLEGGWEGMIAGWHGYTSHGAHGVAVAADLKSTQEAINKITKNLNSLSELEV<br>KNLQRLSGAMDELHNEILELDEKVDDLRADTISSQIELAVLLSNEGIINSEDEHLLALERKLKKMLGPSA<br>VEIGNGCFETKHKCNQTCLDKIAAGTFDAGEFSLPTFDSLNITAASLNDDGLDNHTILLYYSTAASSLAV<br>TLMIAIFVVYMVSRDNVSCSICL
ELISA	B/Brisbane/60/2008	MKAIIVLLMVVTSNADRICTGITSSNSPHVVKTATQGEVNVTGVIPLTTTPTKSHFANLKGTETRGKLCP<br>KCLNCTDLDVALGRPKCTGKIPSARVSILHEVRPVTSGCFPIMHDRTKIRQLPNLLRGYEHIRLSTHNVI<br>NAENAPGGPYKIGTSGSCPNITNGNGFFATMAWAVPKNDKNKTATNPLTIEVPYICTEGEDQITVWGFHS<br>DNEAQMAKLYGDSKPQKFTSSANGVTTHYVSQIGGFPNQTEDGGLPQSGRIVVDYMVQKSGKTGTITYQR<br>GILLPQKVWCASGRSKVIKGSLPLIGEADCLHEKYGGLNKSKPYYTGEHAKAIGNCPIWVKTPLKLANGT<br>KYRPPAKLLKERGFFGAIAGFLEGGWEGMIAGWHGYTSHGAHGVAVAADLKSTQEAINKITKNLNSLSEL<br>EVKNLQRLSGAMDELHNEILELDEKVDDLRADTISSQIELAVLLSNEGIINSEDEHLLALERKLKKMLGP<br>SAVEIGNGCFETKHKCNQTCLDRIAAGTFDAGEFSLPTFDSLNITAASLNDDGLDNHTILLYYSTAASSL<br>AVTLMIAIFVVYMVSRDNVSCSICL
Neutralization	B/Colorado/06/2017	MKAIIVLLMVVTSSADRICTGITSSNSPHVVKTATQGEVNVTGVIPLTTTPTKSHFANLKGTETRGKLCP<br>KCLNCTDLDVALGRPKCTGKIPSARVSILHEVRPVTSGCFPIMHDRTKIRQLPNLLRGYEHVRLSTHNVI<br>NAEGAPGGPYKIGTSGSCPNITNGNGFFATMAWAVPDKNKTATNPLTIEVPYVCTEGEDQITVWGFHSDN<br>ETQMAKLYGDSKPQKFTSSANGVTTHYVSQIGGFPNQTEDGGLPQSGRIVVDYMVQKSGKTGTITYQRGI<br>LLPQKVWCASGRSKVIKGSLPLIGEADCLHEKYGGLNKSKPYYTGEHAKAIGNCPIWVKTPLKLANGTKY<br>RPPAKLLKERGFFGAIAGFLEGGWEGMIAGWHGYTSHGAHGVAVAADLKSTQEAINKITKNLNSLSELEV<br>KNLQRLSGAMDELHNEILELDEKVDDLRADTISSQIELAVLLSNEGIINSEDEHLLALERKLKKMLGPSA<br>VEIGNGCFETKHKCNQTCLDKIAAGTFDAGEFSLPTFDSLNITAASLNDDGLDNHTILLYYSTAASSLAV<br>TLMIAIFVVYMVSRDNVSCSICL

Yamagata Lineage (B-Y)

Use	Strain	Sequence
Vaccine	B/Singapore/INFTT-16-0610/2016	MVVTSNADRICTGITSSNSPHVVKTATQGEVNVTGVIPLTTTPTKSYFANLKGTRTRGKLCPD<br>CLNCTDLDVALGRPMCVGTTPSAKASILHEVRPVTSGCFPIMHDRTKIRQLPNLLRGYEKIRLSTQNVI<br>DAEKAPGGPYRLGTSGSCPNATSKIGFFSTMAWAVPKDNYKNATNPQTVEVPYICTEGEDQITVWGFHSDN<br>KTQMKSLYGDSNPQKFTSSANGVTTHYVSQIGDFPDQTEDGGLPQSGRIVVDYMVQKPGKTGTIVYQRG<br>VLLPQKVWCASGRSKVIKGSLPLIGEADCLHEEYGGLNKSKPYYTGKHAKAIGNCPIWVKTPLKLANGTK<br>YRPPAKLLKERGFFGAIAGFLEGGWEGMIAGWHGYTSHGAHGVAVAADLKSTQEAINKITKNLNSLSELE<br>VKNLQRLSGAMDELHNEILELDEKVDDLRADTISSQIELAVLLSNEGIINSEDEHLLALERKLKKMLGPS<br>AVDIGNGCFETKHKCNQTCLDRIAAGTFNAGEYSLPTFDSLNITAASLNDDGLDNHTILLYYSTAASSLA<br>VTLMLAIFIVYMVSRDNVSCSICL
ELISA/<br>Neutralization	B/Phuket/3073/2013	MKAIIVLLMVVTSNADRICTGITSSNSPHVVKTATQGEVNVTGVIPLTTTPTKSYFANLKGTRTRGKLCP<br>DCLNCTDLDVALGRPMCVGTTPSAKASILHEVRPVTSGCFPIMHDRTKIRQLPNLLRGYEKIRLSTQNVI<br>DAEKAPGGPYRLGTSGSCPNATSKIGFFATMAWAVPKDNYKNATNPLTVEVPYICTEGEDQITVWGFHSDN<br>KTQMKSLYGDSNPQKFTSSANGVTTHYVSQIGDFPDQTEDGGLPQSGRIVVDYMMQKPGKTGTIVYQRG<br>VLLPQKVWCASGRSKVIKGSLPLIGEADCLHEEYGGLNKSKPYYTGKHAKAIGNCPIWVKTPLKLANGTK<br>YRPPAKLLKERGFFGAIAGFLEGGWEGMIAGWHGYTSHGAHGVAVAADLKSTQEAINKITKNLNSLSELE<br>VKNLQRLSGAMDELHNEILELDEKVDDLRADTISSQIELAVLLSNEGIINSEDEHLLALERKLKKMLGPS<br>AVDIGNGCFETKHKCNQTCLDRIAAGTFNAGEFSLPTFDSLNITAASLNDDGLDNHTILLYYSTAASSLA<br>VTLMLAIFIVYMVSRDNVSCSICL
Challenge	B/Florida/04/2006	MKAIIVLLMVVTSNADRICTGITSSNSPHVVKTATQGEVNVTGVIPLTTTPTKSYFANLKGTRTRGKLCP<br>DCLNCTDLDVALGRPMCVGTTPSAKASILHEVKPVTSGCFPIMHDRTKIRQLPNLLRGYENIRLSTQNVI<br>DAEKAPGGPYRLGTSGSCPNATSKSGFFATMAWAVPKDNNKNATNPLTVEVPYICTEGEDQITVWGFHSD<br>DKTQMKNLYGDSNPQKFTSSANGVTTHYVSQIGSFPDQTEDGGLPQSGRIVVDYMMQKPGKTGTIVYQRG<br>VLLPQKVWCASGRSKVIKGSLPLIGEADCLHEKYGGLNKSKPYYTGEHAKAIGNCPIWVKTPLKLANGTK<br>YRPPAKLLKERGFFGAIAGFLEGGWEGMIAGWHGYTSHGAHGVAVAADLKSTQEAINKITKNLNSLSELE<br>VKNLQRLSGAMDELHNEILELDEKVDDLRADTISSQIELAVLLSNEGIINSEDEHLLALERKLKKMLGPS<br>AVEIGNGCFETKHKCNQTCLDRIAAGTFNAGEFSLPTFDSLNITAASLNDDGLDNHTILLYYSTAASSLA<br>VTLMLAIFIVYMVSRDNVSCSICL

Supplementary Fig. S1. Sequences of influenza B viral HA proteins used in this study. Sequences are for influenza B HA in commercial vaccines, and proteins/viruses used in ELISA and neutralization assays and challenge experiments. Sequences were downloaded from different databases such the NCBI GenBank. Influenza B HA of Victoria lineage is denoted as B-V, while Influenza B HA of Yamagata-lineage is denoted as B-Y.

These are all the pairwise sequence identity percentages (from chimera), and also attached is a graphic of the multiple sequence alignment.

AGM53847.1 hemagglutinin [Influenza B virus (B/Florida/04/2006)] vs. AGM53847.1 hemagglutinin [Influenza B virus (B/Florida/04/2006)]: 100.00% identity
AGM53847.1 hemagglutinin [Influenza B virus (B/Florida/04/2006)] vs. ANC28539.1 hemagglutinin [Influenza B virus (B/Brisbane/60/2008)]: 93.49% identity
AGM53847.1 hemagglutinin [Influenza B virus (B/Florida/04/2006)] vs. ASW32353.1 hemagglutinin [Influenza B virus (B/Maryland/15/2016)]: 92.97% identity
AGM53847.1 hemagglutinin [Influenza B virus (B/Florida/04/2006)] vs. AQY15026.1 hemagglutinin [Influenza B virus (B/Iowa/06/2017)]: 93.14% identity
AGM53847.1 hemagglutinin [Influenza B virus (B/Florida/04/2006)] vs. ASK81305.1 hemagglutinin [Influenza B virus (B/Colorado/06/2017)]: 92.97% identity
ANC28539.1 hemagglutinin [Influenza B virus (B/Brisbane/60/2008)] vs. AGM53847.1 hemagglutinin [Influenza B virus (B/Florida/04/2006)]: 93.49% identity
ANC28539.1 hemagglutinin [Influenza B virus (B/Brisbane/60/2008)] vs. ANC28539.1 hemagglutinin [Influenza B virus (B/Brisbane/60/2008)]: 100.00% identity
ANC28539.1 hemagglutinin [Influenza B virus (B/Brisbane/60/2008)] vs. ASW32353.1 hemagglutinin [Influenza B virus (B/Maryland/15/2016)]: 99.14% identity
ANC28539.1 hemagglutinin [Influenza B virus (B/Brisbane/60/2008)] vs. AQY15026.1 hemagglutinin [Influenza B virus (B/Iowa/06/2017)]: 99.14% identity
ANC28539.1 hemagglutinin [Influenza B virus (B/Brisbane/60/2008)] vs. ASK81305.1 hemagglutinin [Influenza B virus (B/Colorado/06/2017)]: 98.97% identity
ASW32353.1 hemagglutinin [Influenza B virus (B/Maryland/15/2016)] vs. AGM53847.1 hemagglutinin [Influenza B virus (B/Florida/04/2006)]: 92.97% identity
ASW32353.1 hemagglutinin [Influenza B virus (B/Maryland/15/2016)] vs. ANC28539.1 hemagglutinin [Influenza B virus (B/Brisbane/60/2008)]: 99.14% identity
ASW32353.1 hemagglutinin [Influenza B virus (B/Maryland/15/2016)] vs. ASW32353.1 hemagglutinin [Influenza B virus (B/Maryland/15/2016)]: 100.00% identity
ASW32353.1 hemagglutinin [Influenza B virus (B/Maryland/15/2016)] vs. AQY15026.1 hemagglutinin [Influenza B virus (B/Iowa/06/2017)]: 99.66% identity
ASW32353.1 hemagglutinin [Influenza B virus (B/Maryland/15/2016)] vs. ASK81305.1 hemagglutinin [Influenza B virus (B/Colorado/06/2017)]: 99.49% identity
AQY15026.1 hemagglutinin [Influenza B virus (B/Iowa/06/2017)] vs. AGM53847.1 hemagglutinin [Influenza B virus (B/Florida/04/2006)]: 93.14% identity
AQY15026.1 hemagglutinin [Influenza B virus (B/Iowa/06/2017)] vs. ANC28539.1 hemagglutinin [Influenza B virus (B/Brisbane/60/2008)]: 99.14% identity
AQY15026.1 hemagglutinin [Influenza B virus (B/Iowa/06/2017)] vs. ASW32353.1 hemagglutinin [Influenza B virus (B/Maryland/15/2016)]: 99.66% identity
AQY15026.1 hemagglutinin [Influenza B virus (B/Iowa/06/2017)] vs. AQY15026.1 hemagglutinin [Influenza B virus (B/Iowa/06/2017)]: 100.00% identity
AQY15026.1 hemagglutinin [Influenza B virus (B/Iowa/06/2017)] vs. ASK81305.1 hemagglutinin [Influenza B virus (B/Colorado/06/2017)]: 99.83% identity
ASK81305.1 hemagglutinin [Influenza B virus (B/Colorado/06/2017)] vs. AGM53847.1 hemagglutinin [Influenza B virus (B/Florida/04/2006)]: 92.97% identity
ASK81305.1 hemagglutinin [Influenza B virus (B/Colorado/06/2017)] vs. ANC28539.1 hemagglutinin [Influenza B virus (B/Brisbane/60/2008)]: 98.97% identity
ASK81305.1 hemagglutinin [Influenza B virus (B/Colorado/06/2017)] vs. ASW32353.1 hemagglutinin [Influenza B virus (B/Maryland/15/2016)]: 99.49% identity
ASK81305.1 hemagglutinin [Influenza B virus (B/Colorado/06/2017)] vs. AQY15026.1 hemagglutinin [Influenza B virus (B/Iowa/06/2017)]: 99.83% identity
ASK81305.1 hemagglutinin [Influenza B virus (B/Colorado/06/2017)] vs. ASK81305.1 hemagglutinin [Influenza B virus (B/Colorado/06/2017)]: 100.00% identity

Supplementary Fig. S2. Sequence comparison and sequence accession numbers of influenza B viral HA proteins used in this study. Accession numbers and strain names are given with the percent identity at the end of each line for the sequence comparison.

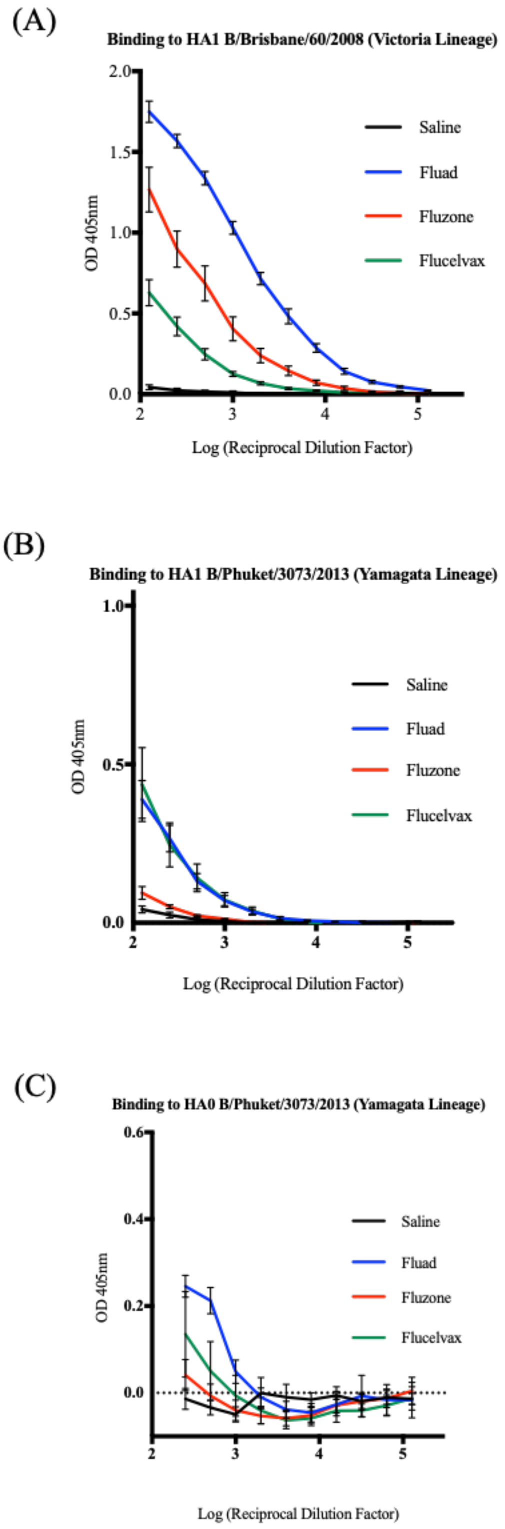

Supplementary Fig. S3. Dilution curves of the antibody binding from vaccinated mouse sera to influenza B HA1 proteins. Serially diluted sera from mice immunized with commercial vaccines Fluad (T), Flucelvax (Q), and Fluzone (T) were analyzed by ELISA for binding to antigenically matched and mismatch influenza HA1 proteins representing (A) B/Brisbane/60/08 (Victoria-lineage), (B) B/Phuket/307/13 (Yamagata lineage) viruses.

(A)

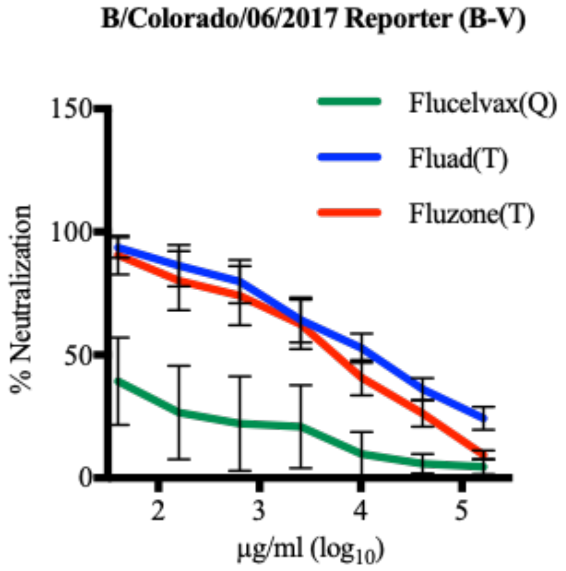

(B)

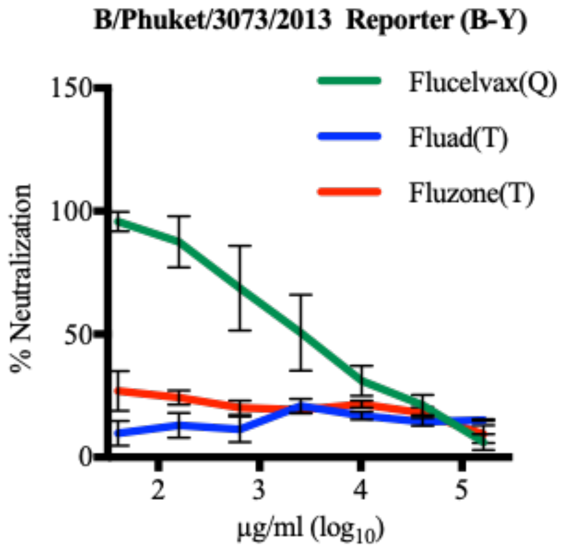

Supplementary Fig. S4. Titration curves for neutralization of influenza B reporter viruses by sera from mice immunized with commercial influenza vaccines. Reporter influenza B viruses were (A) B/Colorado/17 (B-V) and (B) B/Phuket/307/13 (B-Y). Influenza B HA of Victoria lineage is denoted as B-V, while Influenza B HA of Yamagata-lineage is denoted as B-Y.

(A)

Histologic Score	
Bronchiole lumina	Exudate in bronchiole lumina. None (score 0 points), Exudate present (score 1 point); moderate (score 2 points); Severe-partial to complete occlusion of 1 or more central bronchioles (score 3 points)
Terminal bronchiole lumina	Exudate in terminal bronchiolar lumina. None (score 0 points), mild (score 1 point), moderate (score 2 points), Severe-partial to complete occlusion of 1 or more central bronchioles (score 3 points)
Bronchiolar/TB epithelial necrosis/degeneration	None (score 0 points); Mild (score 1 point); Moderate (score 2 points); Severe (score 3 points)
Perivascular inflammation/fibrin	None (score 0 points); Mild (score 1 point); Moderate (score 2 points); Severe (score 3 points)
Alveoli/interstitium edema	None (score 0 points); <25% (score 1 point); 25-50% (score 2 points); >50% (score 3 points)
Alveoli inflammatory cells	None-few (score 0 points); mild increase (score 1 point); moderate (score 2 points); Marked (score 3 points)
Interstitial inflammatory cells	None-few (score 0 points); mild increase (score 1 point); moderate (score 2 points); Marked (score 3 points)
Anywhere: hemorrhage/necrosis/fibrin	None (score 0 points); up to 5% of section (score 1 point); 5-50% of section (score 2 points); >50% of section (score 3 points)

(B)

Influenza B Immunohistochemical Score	
Trachea and bronchiolar epithelium	None (score 0 points), Up to 5% (score 1 point), 5-50% (score 2 points), >50% (score 3 points)
Terminal bronchiolar epithelium	None (score 0 points), Up to 5% (score 1 point), 5-50% (score 2 points), >50% (score 3 points)
Alveolar septa (with immunoreactive interstitial cells) immunoreactivity	None (score 0 points); up to 5% (score 1 point), 5-50% (score 2 points), >50 % (score 3 points)
Bronchiolar exudate	Immunostaining absent (score 0 points); Immunostaining present score 1 point)
Alveolar exudate (+/- immunoreactive alveolar macrophages)	Immunostaining absent (score 0 points); Immunostaining present score 1 point)

Supplementary Fig. S5. Scoring rubrics for pathology of lungs in mice challenged with influenza. (A) Histologic scoring. (B) Influenza B immunohistochemical scoring. Yamagata-lineage challenge virus was mouse-adapted B/Florida/04/2006.
